# Classification of Lung Nodules Based on Deep Residual Networks and Migration Learning

**DOI:** 10.1155/2020/8975078

**Published:** 2020-03-30

**Authors:** Panpan Wu, Xuanchao Sun, Ziping Zhao, Haishuai Wang, Shirui Pan, Björn Schuller

**Affiliations:** ^1^College of Computer and Information Engineering, Tianjin Normal University, Tianjin, China; ^2^Department of Computer Science and Engineering, Fairfield University, Fairfield, USA; ^3^Faculty of Information Technology, Monash University, Clayton, Australia; ^4^Group on Language, Audio & Music, Imperial College London, London, UK; ^5^ZD.B Chair of Embedded Intelligence for Health Care and Wellbeing, University of Augsburg, Augsburg, Germany

## Abstract

The classification process of lung nodule detection in a traditional computer-aided detection (CAD) system is complex, and the classification result is heavily dependent on the performance of each step in lung nodule detection, causing low classification accuracy and high false positive rate. In order to alleviate these issues, a lung nodule classification method based on a deep residual network is proposed. Abandoning traditional image processing methods and taking the 50-layer ResNet network structure as the initial model, the deep residual network is constructed by combining residual learning and migration learning. The proposed approach is verified by conducting experiments on the lung computed tomography (CT) images from the publicly available LIDC-IDRI database. An average accuracy of 98.23% and a false positive rate of 1.65% are obtained based on the ten-fold cross-validation method. Compared with the conventional support vector machine (SVM)-based CAD system, the accuracy of our method improved by 9.96% and the false positive rate decreased by 6.95%, while the accuracy improved by 1.75% and 2.42%, respectively, and the false positive rate decreased by 2.07% and 2.22%, respectively, in contrast to the VGG19 model and InceptionV3 convolutional neural networks. The experimental results demonstrate the effectiveness of our proposed method in lung nodule classification for CT images.

## 1. Introduction

Lung cancer is a malignant disease with poor prognosis, and the average 5-year survival rate of patients is less than 20%. Although there are targeted treatments and various radiotherapy and chemotherapy regimens, the average survival time of advanced lung cancer is only 12 months [1]. Therefore, early detection, early diagnosis, and early treatment of lung cancer can effectively improve the quality of life and survival rate of patients. A spot on the CT of the lung is defined as a lung nodule, which can be benign or malignant. Early lung lesions are mainly characterized by malignant nodules in the lungs. Therefore, it is very important for the clinical treatment of lung cancer to classify lung nodules timely and accurately. In recent years, the improvement of medical and health levels has led to the application of more and more medical digital imaging devices to the clinic. The medical imaging equipment, including X-ray, B-scan ultrasonography, computed tomography (CT), and magnetic resonance imaging (MRI), are improving and optimizing constantly [2]. More and more subtle lesions can be captured by imaging equipment. Among them, computerized tomography is considered to be one of the most effective means of detecting lung cancer early [3]. Doctors need to diagnose malignant nodules accurately by reading the patient's lung CT image; however, reading a large number of CT images is not only time-consuming, and there is also a high probability of misdiagnosis.

With the development of information technology and medical imaging methods, computer-aided detection systems based on CT images have achieved certain research results [4,5]. The use of the CAD system model for the automatic detection and identification of lung nodules not only improves efficiency greatly, it also has higher accuracy and better robustness. The traditional CAD algorithm is divided into two steps: the first step is image preprocessing [6] (pulmonary CT image enhancement, lung parenchymal segmentation, lung ROI extraction, etc.), and the second step is image feature extraction (gray texture features [7], scale-invariant features [8], local binary pattern features [9] and gradient direction histogram features [10], etc.), after which traditional machine learning algorithms (K-nearest neighbors, support vector machines, random forests, etc.) are applied for lung nodule classification. For example, Manikandan and Bharathi [11] extracted grayscale features and the center of gravity of the region of interest for morphological-based target detection classification. Kim et al. [12] extracted 96 nodular morphological features including area, standard deviation, perimeter, and diameter and then extracted 100 ROI deep features with a stacked denoising autoencoder (SDAE) containing 3 hidden layers. Since these features are designed manually, it is difficult to analyze the image comprehensively and deeply. Furthermore, using traditional machine learning algorithms, the previous processing results have a greater impact on the subsequent processing.

As an important branch of machine learning, deep learning has developed rapidly in recent years. Convolutional neural networks (CNNs) have achieved good results in the field of face recognition, object detection, image classification, and other images, due to a large amount of available data and the efficient computing capacity of GPUs, and it has also been applied to medical images [13,14]. Ronneberger et al. [15] reported a new full convolution network (FCN) called U-Net for biomedical image segmentation and achieved promising results. Gao et al. [16] extracted the time domain and spatial domain information features of the echocardiogram by combining two 2D-CNNs and classified the video images of echocardiography to assist diagnosis of heart disease. In 2017, Litjens et al. [17] published a review, which summarizes the research work on deep learning in medical image classification, detection and segmentation, registration, and retrieval.

In the present work, we focus on the research work of lung nodule classification based on a proposed deep residual network. Our two main contributions can be summarized as follows: (1) We have developed a deep residual network based classification method for lung nodules in CT images, abandoning the complex traditional CT image processing method. Our results demonstrate the effectiveness of this approach for lung nodule classification. (2) We also investigated performance comparisons of our approach with two representative deep learning models and one traditional model. It is shown that the performance of our proposed deep learning methods is superior to that of the other two deep learning models and the traditional machine learning method. The presented results indicate the suitability of this approach for lung nodule classification.

The rest of this article is organized as follows. In Section 2, the related work is presented. In Section 3, the related algorithms and method framework are elaborated. The detailed experimental setup, procedure, result comparison, and analysis are presented and discussed in Section 4. The conclusion is given in the last section.

## 2. Related Work

From the literature, it can be observed that deep learning has achieved a series of satisfactory results in the field of medical imaging, and it also has made great progress in the classification of lung nodules. Hua et al. [18] applied CNNs and a deep belief network (DBN) to distinguish malignant from benign lung nodules, and better discriminative results were achieved with deep learning algorithms. A multicrop CNN was developed by Shen et al. [19], and it is able to automatically extract salient nodule information via cropping different regions from convolutional feature maps and applying max-pooling at varying times. Song et al. [20], respectively, adopted the LeNet (4-layer) CNN structure, a deep neural network (DNN), and a stacked autoencoder (SAE) for lung nodule classification. The CNN network achieved the best performance with an accuracy of 84.2% on the LIDC-IDRI lung image dataset. Hussein et al. [21] proposed an end-to-end deep multiview CNN based on the AlexNet (8-layer) network structure and achieved 92.3% classification accuracy of lung nodules on the LIDC-IDRI dataset. Nibali et al. [22] combined a residual network, course learning, and migration learning to propose the ResNet-18 network structure for lung nodule classification. A classification accuracy of 89.9% was obtained on the test samples collected from the LIDC-IDRI dataset.

Traditional computer-aided detection algorithms based on machine learning have the problem of low classification accuracy due to the uncertainty of artificial feature selection. Most classes handled by CNNs are more obvious classes (such as humans and dogs), and there are only subtle differences between the data in the nodule classification task, so there are certain disadvantages in using CNNs for lung nodule classification tasks. The residual network proposed by He et al. [23] in 2015 won as the champion of the ImageNet Large Scale Visual Recognition Competition (ILSVRC) [24]. As a deep learning model, the residual network has been applied successfully in the fields of text classification and image classification. Lan et al. [25] proposed a new network called Residual U-Net (RUN) to perform the lung nodule detection without the selection of nodule candidates. The idea of a residual network was introduced to improve the traditional U-Net, thus solving the shortcomings of poor results from lacking of network depth. Migration learning is also a very useful machine learning method, which means a pretrained model is reapplied to another task. Using the pretrained CNN models on ImageNet for migration learning has become a common method in medical image analysis. van Ginneken et al. [26] extracted the 4096 off-the-shelf features from the first fully connected layer of a pretrained OverFeat model for lung nodule detection in CT images through a linear support vector machine (SVM).

Inspired by the aforementioned work, in this paper, combined with the theories of residual learning and migration learning, a classification method for lung nodules based on a deep residual network is proposed. The 50-layer ResNet network structure is used as the initial model to reconstruct the global average pooling layer, the fully connected layer, and the classification layer. The experimental results on the LIDC-IDRI dataset show that the proposed method has better performance and adaptability than the SVM method, VGG19 model [27], and InceptionV3 model [28] in the classification of lung nodules.

## 3. Algorithm and Method Framework

**Figure 1:**
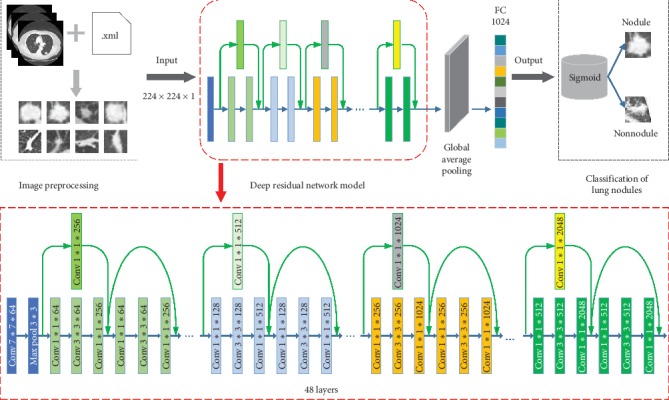
Classification model of pulmonary nodules based on deep residual network.

In the present work, the basic principle of a residual network is applied to the classification of pulmonary nodules. The proposed classification method of pulmonary nodules based on a deep residual network includes: (1) on the LIDC-IDRI dataset, the nodule contour is extracted according to the labeling of the experienced radiologists to form the experimental dataset; (2) based on the 50-layer residual network and migration learning idea, the original network weight is preserved, and the global average pooling layer, fully connected layer, and classification layer are constructed; (3) the network model is trained using the extracted experimental dataset to complete the classification task of the pulmonary nodules.  Figure 1 depicts the pulmonary nodule classification process based on a deep residual network.

### 3.1. Residual Learning

In the process of deep learning, the main problems with an increase in network depth are gradient disappearance and gradient explosion. The traditional solution is the initialization and regularization of data, which solves the gradient problem but results in network performance degradation. The depth has deepened, but the error rate has increased. The purpose of residual learning is to improve the network performance while solving the network gradient problem. If the layers behind the deep network are all identically mapped, the model can be degraded into a shallow network, and then, the problem of network performance degradation caused by the increase in the network depth can be solved.

**Figure 2:**
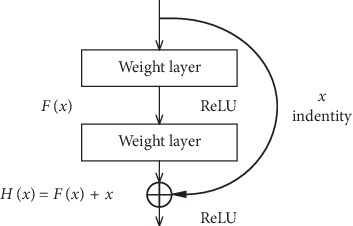
Residual element.

To achieve identity mapping, only the identity mapping function needs to be used. In residual learning, we design the network as *H*(*x*)=*F*(*x*)+*x*, thus, it is converted to learn a residual function *F*(*x*)=*H*(*x*) − *x*, and as long as the fitting *F*(*x*)=0, it forms an identity map *H*(*x*)=*x*, where *x* represents the prelayer input, *F*(*x*) is the network mapping before the sum, and *H*(*x*) is the network mapping from the input to summation.  Figure 2 depicts the residual elements in the residual network, which is a basic building block in the residual network.

Considering the forward process, the final result represents a direct forward process from the *l* layer to the *L* layer and is a continuous operation. The specific calculation process is as follows:

(1) $$\begin{array}{c} x_{l+1}=x_{l}+F\left(x_{l},W_{l}\right)\\ x_{l+2}=x_{l+1}+F\left(x_{l+1},W_{l+1}\right)\\ =x_{l}+F\left(x_{l},W_{l}\right)+F\left(x_{l+1},W_{l+1}\right)\\ \ldots \ldots \\ x_{L}=x_{l}+\sum_{i=l}^{L-l}F\left(x_{i},W_{i}\right), \end{array}$$

where *W*_*i*_ represents the equivalent mapping method. For residual elements, the forward process is linear, and the subsequent input is equal to the result of the input plus each residual element. The first major feature of the residual network is that the reverse update solves the problem of gradient disappearance. Therefore, when the residual network propagates in the back direction, only the part before the chain law is derived, that is, the gradient from the *L* layer can be transferred to the *l* layer stably. The specific derivation process is

(2) $$\begin{array}{c} \frac{\partial E}{\partial x_{l}}=\frac{\partial E}{\partial x_{L}}\frac{\partial x_{L}}{\partial x_{l}}=\frac{\partial E}{\partial x_{L}}\left(1+\frac{\partial }{\partial x_{l}}\sum_{i=l}^{L-1}F\left(x_{i},W_{i}\right)\right). \end{array}$$

Through the structural design of the residual element, the network will avoid the problem of complete gradient disappearance when performing backpropagation training. At the same time, when the performance of the network reaches a bottleneck, the redundant network layer can do identical mapping, which realizes the basic idea of residual learning.

### 3.2. Deep Residual Network Based Pulmonary Nodule Classification Methodology

As outlined, the lung nodule classification method of deep residual networks proposed in this paper is based on the 50-layer ResNet network, retaining the original weights trained on the ImageNet dataset, removing the original fully connected and classification layers of the network, and adding the global average pooling layer. The global average pooling layer was originally used by Min Lin and Yan in 2014 [29]. They used global average pooling to replace the traditional fully connected layers in the CNN. The idea was to generate one feature map for each corresponding category of the classification task in the last mlpconv layer. They took the average of each feature map, and the resulting vector was fed directly into the softmax layer. Since there are many parameters for the fully connected layer and the fully connected layer are prone to overfitting, thus hampering the generalization ability of the overall network. The number of parameters is reduced greatly after adding the global average pooling layer, which can compress the size of the model well and reduce the occurrence of overfitting. Furthermore, global average pooling sums out the spatial information, thus it is more robust to spatial translations of the input.

After global average pooling layer, we reconstruct the fully connected layer and the classification layer. Since differentiating nodules from nonnodules is a binary classification problem, the sigmoid is used in the classification layer. The ReLU function is employed as an activation function, which makes the feature extraction range of neurons more extensive. The formula is

(3) $$\begin{array}{c} \text{ReLU}\left(x\right)=\left\{\begin{array}{cc} x, & \text{if }x>0,\\ 0, & \text{if }x\leq 0. \end{array}\right. \end{array}$$

The design of the deep residual network is embodied by the implementation of the identity mapping in the fast connection mode. The fast connection makes the residual possible, and the identity mapping makes the network deeper. The quick connection can be embodied as our residual network and is composed of multiple stacked layers, each of which uses the residual element as shown in Figure 2. Specifically, the building block of this article is defined as

(4) $$\begin{array}{c} y=F\left(x,\left\{W_{i}\right\}\right)+x, \end{array}$$

where *x* and *y* represent the input and output vectors and *W*_*i*_ represents the equivalent mapping method. The function *F*(*x*, {*W*_*i*_}) denotes the residual function. As shown in Figure 2, there are two layers, *F*=*W*_2_*σ*(*W*_1_*x*), in which *σ* represents ReLU. For simplicity, we temporarily ignore the offset. The operation of *F*+*x* is done by a quick connection and an element-by-element addition.

In the design process of this method, the identity maps are implemented by shortcut connections and their outputs are added to the output of the overlay. Shortcut connections neither generate additional parameters nor add computational complexity, and the entire network still uses the backpropagated stochastic gradient descent (SGD) algorithm. At the same time, the residual structures can connect the fully connected layers of the network with the features of each layer of the lung nodule images indirectly, which merge the shallow features and deep features of the image effectively. The residual network can exploit the useful information contained in the images fully, which is expected to improve the accuracy of lung nodule classification [30].

## 4. Experiment and Result Analysis

### 4.1. Datasets and Preprocessing

The lung CT images used in our experiment are from the Lung Image Database Consortium collection (LIDC-IDRI) [31–33]. Currently, the LIDC-IDRI dataset is the world's largest public dataset for lung cancer and contains 1,018 cases (a total of 375,590 CT scan images with a scan layer thickness of 1.25 mm∼3 mm and 512 × 512 pixels). For each subject, the nodules are identified by four experienced thoracic radiologists without forced consensus, and the corresponding outline coordinates and characteristic information of the nodules are recorded in an associated XML file. In our experiment, except 8 missing cases and one with damaged images, a total of 1009 instances are adopted to evaluate the effectiveness of the proposed method.

**Figure 3:**
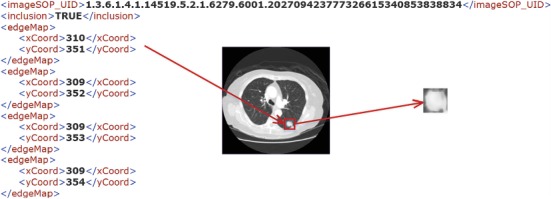
Illustration of extracting lung nodule region from CT images.

In the process of pulmonary nodule detection, the lung nodule area is initially extracted from the lung CT image. The traditional processing approaches are divided into several procedures such as image preprocessing and image feature extraction. These steps are commonly set by humans, and the result of each step has a direct influence on the subsequent classification performance. Therefore, in the present work, we abandon the traditional method of lung nodule extraction, and according to the radiologist's annotations of the pulmonary nodules, the pulmonary nodule area is extracted directly as the experimental dataset. The size of the lung nodules is in the range of 3 mm∼30 mm, and the nodule area on each CT image is marked by the radiologists. According to the nodule contour coordinate information marked by the radiologist in the associated XML file, the rectangular region of the nodule can be segmented from the CT images, as shown in Figure 3. In addition, the nonnodules ≥3 mm are extracted based on their centroids annotated by the radiologists.

**Figure 4:**
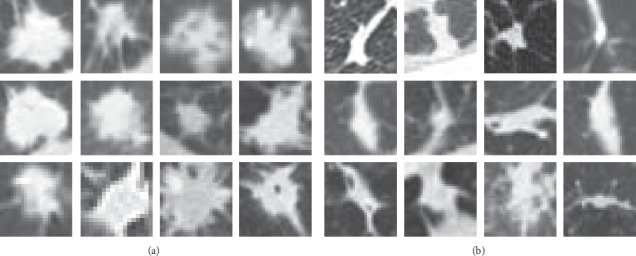
Illustration of extracting lung nodule region from CT images: (a) nodule samples. (b) nonnodule samples.

In our experiment, a total of 14995 CT slices are collected from 1009 instances in terms of the radiologist's annotations, including 7685 nodule slices and 7310 nonnodule slices. The nodule and nonnodule samples are shown in Figure 4.

### 4.2. Experimental Setup

In order to validate the effectiveness of the network model, the accuracy, precision, specificity, recall, f1-score, false positive rate, and receiver operating characteristic (ROC) curve of the classification of pulmonary nodules are used to evaluate the performance of the algorithm in this paper. All the possible outcomes of a test procedure and the gold standard are listed in Table 1.  Table 2 details the formulas to calculate the aforementioned evaluation indexes. Additionally, a statistical significance test is performed to observe the performance differences of different approaches.

All the experiments were evaluated using ten-fold cross-validation, and the validation dataset and test dataset were swapped to repeat the experiment. The average result is taken as the final experiment result. The data volume of the nodules and nonnodules is equal basically to confirm the effectiveness of the network. These two hyperparameters of the optimizer SGD method are set to: learning rate (LR) = 0.0001 and momentum = 0.9 [34]. Because the model requires that the input size of the image is 224 × 224 × 1, the extracted nodule images are resized to the same size before being input into our model.

In order to show the effectiveness of our proposed approach, we compare the lung nodule classification performances with different models, namely, the traditional machine learning model and the pretrained CNN models. Specifically, the SVM classification model with curvelet transform features, the VGG19 model and the InceptionV3 model are designed as the comparison algorithms. Among them, the curvelet transform is a multiscale, directional feature extraction method. It has obvious advantages in the description of the contour and texture direction of an image. The fast discrete curvelet transform includes two algorithms. One is the unequispaced FFT transform, in which the curvelet coefficients are found by irregularly sampling the Fourier coefficients of an image. Another one is the Wrapping transform, using a series of translations and a wraparound technique. The unequispaced FFT transform was used in the present work. The first layer (low frequency coefficients) of the curvelet transform coefficients mainly contains the energy of the image and the contour characteristics. The higher frequency coefficients correspond to the image edge and details information. The medium high-level coefficients also describe the edge features. We concatenated all layer coefficients, whose dimension is 4096, as the feature vector. Then, PCA technique was used on it to reduce the feature size by selecting 100 components which explained >95% variances of the original features. The radial basis kernel function is used in the SVM classification method. The penalty parameter *c*=1 of the error term, and the kernel parameter *γ*=0.5. The VGG19 model and the InceptionV3 model contain 16 convolutional layers and 47 convolutional layers, respectively. Detailed configurations of the deep residual network, VGG19 model, and InceptionV3 model are listed in Table 3. The global average pooling layer is also employed in VGG19 and InceptionV3 models. Then, the fully connected layer and the sigmoid classification layer are followed. The collected dataset is used to fine-tune these networks. The same training dataset, verification dataset, and test dataset are used in all four models.

### 4.3. Experimental Results and Analysis

**Figure 5:**
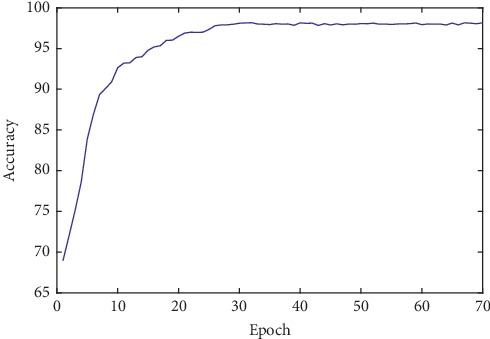
Influence of the classification accuracy with the increase of epoch.

During the training process of the neural network, when all the training datasets are used to train the network once, it is called one epoch. In order to observe the performance relationships between the classification accuracy of the test dataset and the training times, we drew the change curve of the classification accuracy with the increase of the epoch at an interval of 1.  Figure 5 shows the change curve of the classification accuracy with the increase of the epoch.

It can be seen from Figure 5 that accuracy increases with the gradual increase of the epoch. When the epoch reaches 30, the highest accuracy is obtained. As the epoch continues to increase, the accuracy rate remains relatively stable. In line with the experimental setup, the SVM algorithm with curvelet transform features, the VGG19 model, and the InceptionV3 model is compared with the proposed method. A comparison of the classification results of the lung nodules using different methods is shown in Table 4.

Table 4 shows the results of the four methods. As mentioned, the results are obtained based on ten-fold cross-validation. The traditional machine learning reference method, the SVM algorithm, achieves an accuracy of 88.27%, precision of 90.12%, recall of 87.69%, specificity of 85.94%, f1-score of 88.89%, and a false positive rate of 8.60%. The deep learning method, the VGG19 model, achieves an accuracy of 96.48%, precision of 97.10%, recall of 95.17%, specificity of 96.83%, f1-score of 96.13%, and a false positive rate of 3.72%. InceptionV3 achieves an accuracy of 95.81%, precision of 96.35%, recall of 95.30%, specificity of 95.76%, f1-score of 95.85%, and a false positive rate of 3.87%. The lung nodule classification method based on the proposed deep residual network achieves an accuracy of 98.23%, precision of 98.46%, recall of 97.70%, specificity of 98.35%, f1-score of 98.06%, and a false positive rate of 1.65% in our study. Our proposed method outperforms the SVM algorithm by 9.96%, 8.34%, 10.01%, 12.41%, and 9.17% in terms of accuracy, precision, recall, specificity, and f1-score, and the false positive rate is decreased by 6.95%. Our method outperforms the VGG19 model by 1.75%, 1.36%, 2.53%, 1.52%, and 1.93% in terms of accuracy, precision, recall, specificity, and f1-score, and the false positive rate decreased by 2.07%. Furthermore, our method improved by 2.42%, 2.11%, 2.40%, 2.59%, and 2.21% in terms of accuracy, precision, recall, specificity, and f1-score, respectively, compared to the InceptionV3 model, and the false positive rate decreased by 2.22%. This represents a significant improvement over the SVM model (*p* < 0.01 in a one-tailed z-test), VGG19 model (*p* < o.01 in a one-tailed z-test), and InceptionV3 model (*p* < 0.01 in a one-tailed z-test).

**Figure 6:**
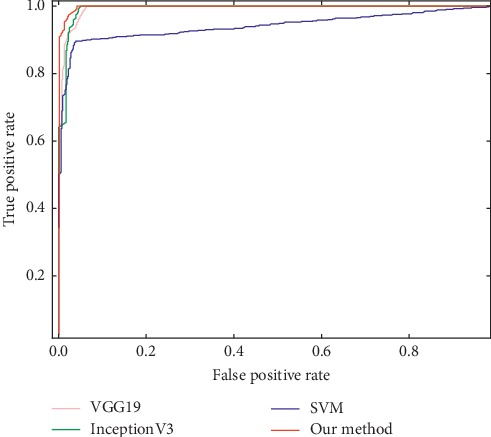
ROC curves of different classification methods.

The ROC curve and the area under the ROC curve (AUC) are important indicators for evaluating the performance of the algorithm. The closer the ROC curve is to the upper left, the closer the AUC value is to 1, indicating that the classification result of the algorithm is better. In order to facilitate an intuitive comparison, the ROC curves of the different methods are drawn in a unified coordinate graph, as shown in Figure 6.

As can be seen in Figure 6, the ROC curve of our method is closer to the upper left of the graph than the other three methods. Comparable results are achieved by our method, the VGG19 model, and the InceptionV3 model upon the AUC value. A relatively lower result is got by the SVM model. The corresponding AUC values are our method of 0.9971, VGG19 of 0.9935, InceptionV3 of 0.9924, and SVM of 0.9414, respectively. Again, this represents a significant improvement over the SVM (*p* < 0.01 in a one-tailed z-test) and InceptionV3 model (*p* < 0.05 in a one-tailed z-test). Although no significant performance differences (*p* < 0.05) are observed between our method and VGG19 model, it is evident that our method is superior to VGG19 model (*p* < 0.01 in a one-tailed z-test) in terms of the ROC curve and other evaluation criteria mentioned above. Overall, better results are obtained with our method, which further verifies the feasibility and validity of the proposed method.

Given these experimental results, we can see that the proposed residual network achieves better classification results. The accuracy, precision, recall, f1-score, false positive rate, and AUC values of lung nodule classification are higher than that of the SVM method, the VGG19 model, and the InceptionV3 model. In addition, we compare the experimental results of our method with that of several representative neural network models in the work presented in [20–22]. However, it is difficult to make an objective comparison with the previously published literature due to the variability in the dataset and different validation methods. Nevertheless, it is still important to attempt a relative comparison. For this purpose, we identified several representative methods that have used the same dataset (LIDC-IDRI), employed the same validation procedure (ten-fold cross-validation), and reported better results. Thus, we compared our experimental results with those of literatures [20–22]. 4-layer and 8-layer convolutional neural networks are adopted in [20,21], respectively. Both of them use the ten-fold cross-validation method. An 18-layer ResNet network is adopted in [22], and the literature also uses the randomized splits to evaluate the accuracy of models. A comparison of the classification results of lung nodules in different studies is shown in Table 5.

From Table 5, we can observe that our network has the best classification performance, with an accuracy of 98.23%, sensitivity of 97.70%, and specificity of 98.35%. The accuracy, sensitivity, and specificity of our method are higher than those of the other three methods. Furthermore, the traditional machine learning method needs different feature extraction methods to select and extract the different features of lung nodules, which increases the complexity and error rate of the operation. Furthermore, it is difficult to reach a deep layer for traditional neural networks due to the problem of gradient disappearance. However, for our proposed method, because the ResNet network applies the theory of residual learning, the problem of network performance degradation caused by the increase of network depth is settled. Our method can learn the characteristics of lung nodule images automatically, and the number of network layers can reach a certain depth, which has better adaptability to lung nodule classification tasks.

## 5. Conclusion

To address the problems of lung nodule classification, such as a complex classification detection process, low classification accuracy, and high false positive rate, combining the theories of deep learning, migration learning, and residual learning, a novel neural network model for lung nodule classification is proposed. The model is based on a 50-layer residual network model framework, reconstructing the global average pooling layer, the fully connected layer, and the classification layer. The lung nodule image can be used as the input data of the network directly, avoiding complicated feature extraction and selection. The experiment results on the LIDC-IDRI dataset show that the accuracy, precision, specificity, recall, f1-score, false positive rate, and ROC curves of our method outperform the reported results of all the other methods mentioned in this paper, including the neural network models and a traditional machine learning algorithm. This study demonstrates the superior performance of the proposed method in lung nodule classification, which might have the potential to provide a reference for clinical diagnosis.

Although the residual network structure proposed in this paper has better performance in the classification task of lung nodules, it also has a deficiency, this being that a long training time is needed when dealing with a large number of lung CT images. Therefore, it is necessary to further optimize the network model in follow-up work.

## Figures and Tables

**Table 1:** All possible outcomes of a test.

Test result	Gold standard	
	Positive	Negative
Positive	True positive (TP)	False positive (FP)
Negative	False negative (FN)	True negative (TN)
Total	TP + FN	FP + TN

**Table 2:** Indicators for evaluating algorithm performance.

Evaluation criteria	Calculation method
Accuracy	Accuracy = (TP + TN)/(TP + TN + FP + FN)
Precision	Precision = TP/(TP + FP)
Specificity	Specificity = TN/(TN + FP)
False positive rate	FPR = FP/(FP + TN)
Recall	Recall = TP/(TP + FN)
F1-score	F1-score = 2 × precision × recall/(precision + recall)

**Table 3:** Parameter configuration of the deep residual network, VGG19 model, and InceptionV3 model.

Deep residual network	VGG19	InceptionV3	
Input: nodule/nonnodule images			
conv1 7 × 7, 64	2 × conv3-64	conv3-32	
		conv3-32	
		conv3-64	
max pool	max pool	max pool	
conv2_x $\left[\begin{array}{c} 1\times 1,64\\ 3\times 3,64\\ 1\times 1,256 \end{array}\right]\times 3$	2 × conv3-128	conv1-80	
		conv3-192	
	max pool	max pool	
conv3_x $\left[\begin{array}{c} 1\times 1,128\\ 3\times 3,128\\ 1\times 1,512 \end{array}\right]\times 4$	4 × conv3-256	block1	module1 ⟶ concat
			module2 ⟶ concat
	max pool		module3 ⟶ concat
conv4_x $\left[\begin{array}{c} 1\times 1,256\\ 3\times 3,256\\ 1\times 1,1024 \end{array}\right]\times 6$	4 × conv3-512	block2	module1 ⟶ concat
			module2 ⟶ concat
			module3 ⟶ concat
			module4 ⟶ concat
	max pool		module5 ⟶ concat
conv5_x $\left[\begin{array}{c} 1\times 1,512\\ 3\times 3,512\\ 1\times 1,2048 \end{array}\right]\times 3$	4 × conv3-512	block3	module1 ⟶ concat
			module2 ⟶ concat
	max pool		module3 ⟶ concat
Global average pooling2D			
Fully connected layer-1024			
Fully connected layer-2			
Output: sigmoid			

**Table 4:** Comparison of the classification results of lung nodules with different methods.

Methods	Accuracy (%)	Precision (%)	Recall (%)	Specificity (%)	F1-score (%)	FPR (%)
Curvelet + SVM	88.27	90.12	87.69	85.94	88.89	8.60
VGG19	96.48	97.10	95.17	96.83	96.13	3.72
InceptionV3	95.81	96.35	95.30	95.76	95.85	3.87
Deep residual network	**98.23**	**98.46**	**97.70**	**98.35**	**98.06**	**1.65**

**Table 5:** Comparison of classification results of lung nodules in the literature.

Methods	Network layer	Accuracy (%)	Recall (sensitivity)(%)	Specificity (%)
CNN [20]	4	84.20	84.00	84.30
High-level attributes + CNN [21]	8	92.30	—	—
ResNet [22]	18	89.90	91.10	88.60
Deep residual network	**50**	**98.23**	**97.70**	**98.35**

Note:“—” in the table indicates no data.

## Data Availability

Copies of the Lung Image Database Consortium data can be obtained free of charge from https://wiki.cancerimagingarchive.net/display/Public/LIDC-IDRI/.
